# Network Analysis of Key Instrumental Activities of Daily Living and Cognitive Domains for Targeted Intervention in US Older Adults Without Dementia: Cross-Sectional Study

**DOI:** 10.2196/67632

**Published:** 2025-03-19

**Authors:** Jiaying Li, Rendong He, Erh-Chi Hsu, Junxin Li

**Affiliations:** 1 School of Nursing, Johns Hopkins University, 525 N Wolfe St, Baltimore, MD, 21205, United States, 1 4105022608; 2 School of Nursing, Li Ka Shing Faculty of Medicine, University of Hong Kong, China (Hong Kong); 3 School of Nursing, Jilin University, Changchun, China

**Keywords:** cognition function, older adults, intervention targets, elder, elderly, cognitive impairment, stimulating activity, instrumental activities of daily living, IADL, daily living activity, cognitive domain, non-demented, cognitive network, holistic cognition, holistic cognition function, network comparison, central variables, bridge variables, network analysis

## Abstract

**Background:**

Cognitive impairment in older adults reduces independence and raises health care costs but can be mitigated through stimulating activities. Based on network theory, intricate relationships within and between clusters of instrumental activities of daily living (IADLs) and cognitive domains suggest the existence of central IADLs and cognitive domains, as well as bridge IADLs. Modifying these can significantly enhance daily living activities and cognitive functions holistically.

**Objective:**

This study aims to identify central IADLs (key activities within the IADL network), central cognitive domains (key domains within the cognitive network), and bridge IADLs (linking IADL and cognitive networks). These insights will inform targeted interventions to effectively improve IADL and cognitive well-being in older adults.

**Methods:**

A cross-sectional analysis of adults aged 65 years and older in the United States focused on 5 IADLs and 6 cognitive domains from the National Health and Aging Trends Study (NHATS). Network analysis identified central and bridge variables. Nonparametric and case-dropping bootstrap methods checked network stability. Network comparison tests assessed sex differences with Benjamini-Hochberg adjustments.

**Results:**

Of the 2239 participants, 56.4% were female (n=976). We computed and tested 3 networks: IADL, cognition, and bridge-with correlation stability coefficients of 0.67, 0.75, and 0.44, respectively (all>0.25). Meal preparation was identified as the central IADL, with a centrality index of 3.87, which was significantly higher than that of other IADLs (all *P*<.05). Visual attention emerged as the central cognition domain, with a centrality index of 0.86, which was significantly higher than that of other cognition domains (all *P*<.05). Shopping was determined to be the bridge IADL, with a centrality index of 0.41, which was significantly higher than that of other IADLs (all *P*<.05). Notably, gender differences emerged in the IADL network, with stronger associations between laundry and meal preparation in females (1.69 vs males: 0.74; *P*=.001) and higher centrality in meal preparation among females (difference=1.99; *P*=.007).

**Conclusions:**

While broad enhancements in all IADL and cognitive domains are beneficial, targeting meal preparation, visual attention, and shopping may leverage their within-network influence to yield a more pronounced improvement in holistic IADL, holistic cognition, and holistic cognition function through IADL interventions among older adults. Notably, meal preparation interventions may be less effective in males, requiring tailored approaches.

## Introduction

Cognitive function encompasses mental processes such as acquiring knowledge, manipulating information, and reasoning, including perception, memory, learning, attention, decision-making, and language abilities [1]. Globally, cognitive impairment in older adults has a prevalence ranging from 5.1% to 41%, with incidence rates around 53.97 per 1000 person-years [2]. This impairment not only predicts older adults’ future incidence of dementia, but also significantly reduces functional independence and quality of life [2]. Beyond the individual level, economically, cognitive impairment incurs 44% higher direct medical costs and significantly increases the need for informal care [3]. Given these consequences, early detection and proactive management are essential to mitigate impacts and prevent progression to more severe conditions and greater health care burdens.

Instrumental activities of daily living (IADLs) encompass complex tasks necessary for independent living, such as cooking, cleaning, transportation, laundry, and financial management [4]. These activities assessment sometime adjusted by country or age will include driving or medication management [5,6] and demand cognitive skills like planning, memory, and problem-solving, linking their performance closely to cognitive well-being [4]. In performing these tasks, individuals engage in practical cognitive training that builds cognitive reserve and supports overall brain health. Furthermore, regularly performing IADLs provides ongoing cognitive training that builds “cognitive reserve,” a concept suggesting that mentally stimulating tasks strengthen neural connections, thereby enhancing brain resilience [7]. Although other pursuits, such as employment or volunteering, also require high-level cognition, IADLs foster daily independence in older adults, and their everyday, repetitive occurrence makes them an ideal approach for continuous cognitive training. However, current interventions usually target specific IADL domains, such as shopping or meal preparation, highlighting the challenges and resource demands of broad IADL interventions [8].

A significant research gap exists in identifying the most impactful IADL component that can efficiently enhance global cognitive function, particularly crucial in resource-limited settings. This need is supported by complex interactions between IADLs and cognition [9-12], correlations within individual IADL domains (eg, between laundry and meal preparation due to similar cognitive and physical demands) [13], and relationships within cognition domains (between psychomotor function and visual attention stemming from their joint role in tasks requiring quick coordination and responses) [14]. Notably, the cognitive demands of specific IADLs vary; for instance, shopping necessitates skills in navigation, selection, and financial transactions, whereas meal preparation involves planning, execution, and presentation. Such distinctions suggest the existence of certain IADLs that are more closely linked to multiple cognitive domains. Traditional analytic methods, which often isolate relationships or assume predictor independence, may overlook the nuanced, simultaneous interactions among nodes. In contrast, network analysis captures these complex dynamics by representing variables as nodes interconnected by edges [15]. This approach not only elucidates direct interactions but also reveals broader network structures, thereby identifying “central” nodes-those exerting significant within-group influence-and “bridge” nodes that connect disparate networks [15]. Specifically, within our framework, a central IADL is defined as the activity with the highest connectivity within the IADL network, while central cognition refers to the cognitive domain with the most extensive links. A bridge IADL, by linking the IADL and cognitive networks, may have an outsized impact on overall function when its performance changes. Such insights can inform target interventions and strategic resource allocation aimed at enhancing both daily living activities and cognitive function [15]. Furthermore, gender-specific differences in cognitive decline and IADL performance further complicate this landscape. Women generally demonstrate superior executive function and memory; however, their executive function appears to decline more rapidly than that of men, while memory trajectories remain similar between sexes. In contrast, difficulties in financial management and medication adherence are more predictive of dementia in men, whereas transportation challenges serve as stronger indicators in women [16,17]. Consequently, a nuanced exploration of these differences is critical for developing targeted intervention strategies.

This study aimed to identify the central IADL and cognition domains within their respective networks, pinpoint the bridge IADL most substantially linked to overall cognitive function, and examine sex differences in these variables. We hypothesized the existence of central, bridge variables and sex-based differences affecting them. The findings are expected to reveal network dynamics, pinpoint key intervention targets for effectively enhancing holistic IADL and cognitive functions in the elderly, and indicate the necessity of sex-specific interventions.

## Methods

### Study Design and Data Source

This cross-sectional analysis used waves 11 and 12 (2021‐2022) of the National Health and Aging Trends Study (NHATS), a nationally representative longitudinal database of Medicare beneficiaries aged 65 years and older. Waves 11 and 12 of NHATS were chosen for their comprehensive 6-domain cognitive assessment, unlike earlier waves that measured only episodic memory, executive function, and orientation. Data were gathered during in-home interviews by NHATS interviewers. For those included in both waves, only data from wave 12 were retained to ensure the latest cognitive assessments were used. The reporting of this study followed the CHERRIES checklist [18].

### Participants and Sample Size Calculation

Eligible participants were cognitively intact individuals aged 65 years and older, not residing in nursing homes, and without signs of cognitive impairment. Cognitive intactness was determined by absence of potential or probable dementia. According to previous NHATS literature, potential dementia was defined by scores≤1.5SDs below the mean in one cognitive domain, while probable dementia was indicated by similar scores in at least two domains, meeting AD8 criteria, or having a clinical dementia diagnosis [19]. To ensure adequate statistical power, our network analysis of 11 nodes and 55 edges required a minimum of 165 participants, adhering to the 3-participants-per-parameter rule [20].

### Measures

Cognitive performance was assessed across six domains: (1) Episodic memory, scored 0‐20 from immediate and delayed recall of 10 words; (2) executive function, scored 0‐5 by the clock drawing test; (3) orientation, scored 0‐8 from knowledge of the current date and names of the president or vice president; (4) psychomotor function, measured by reaction speed in log-transformed milliseconds from the cogstate detection task, where participants respond when a card turns face up; (5) visual attention, assessed by reaction speed for correct responses in log-transformed milliseconds from the cogstate identification task, where participants decide if a card is red or black; and (6) working memory, evaluated by accuracy in the cogstate one card back task, asking participants to remember if they have seen a card before. Cogstate, a tablet-based test used since wave 11 in NHATS, includes card detection, identification, and one card back activities, expanding cognitive assessments beyond traditional tests. This computerized assessment has been validated against traditional paper-based cognitive tests, demonstrating adequate reliability and validity in differentiating adults with cognitive impairment from those without [21]. Further details are available in the NHATS Cogstate user guide [22].

IADLs were assessed via self-reports on managing medication, laundry, shopping, meal preparation, and banking. Participants reported performance over the past month using 5 options: “1” Did not do by self last month; “2” Did by self last month with no difficulty; “3” Did by self last month with difficulty; “4” -Don’t know or refuse, with no difficulty; and “5” Don’t know or refuse, with difficulty. We dichotomized responses rather than using a Likert scale because the options do not form a natural continuum, but instead distinguish independent performance from any difficulty. Participants with no difficulty (responses “2” or “4”) were classified as “no difficulty,” while those with difficulty or inability (responses “1,” “3,” or “5”) were classified as “difficulty.” This binary approach preserves the key distinction in functional independence and aligns with previous research [23].

Sociodemographic variables included age (70‐74, 75‐79, 80‐84, 85‐89, and 90+ years), sex (female or male), living arrangement (alone, with others), race (Hispanic, non-Hispanic Black, non-Hispanic White, and other non-Hispanic), income status (poverty, low income, and normal), and self-rated health (poor, fair, good, very good, and excellent). Income status was defined according to federal guidelines from the Office of the Assistant Secretary for Planning and Evaluation at the US Department of Health and Human Services (ASPE HHS, 2024 version) as follows:≤100% Federal Poverty Level (FPL), >100% to≤200% FPL, and>200% FPL, replacing the previous labels of “poverty,” “low income,” and “normal.” Self-rated health was measured using a single 5-point Likert-scale item (1=poor, 5=excellent): “Would you say that, in general, your health is poor, fair, good, very good, or excellent?”

### Statistical Analysis

Data were organized in a Microsoft Excel database and subjected to rigorous quality control checks. The analysis was carried out using R statistical software (version 4.1.1; R Core Team). Descriptive statistics summarized participant demographics and performance in IADLs and cognitive functions. Continuous variables were checked for normality with P-P plots and described using mean and SD; categorical variables were presented as frequencies and percentages. To maintain consistency across all nodes within the IADL and cognition networks, necessary reverse coding adjustments were made to ensure that higher scores consistently indicate diminished capabilities. Network analysis proceeded through 5 phases: evaluating topological overlap, estimating the network, assessing network stability, calculating centrality and bridge centrality indices, and conducting network comparison tests.

#### Checking Topological Overlap

We used the R package *network tools*’ goldbricker function to identify unique variables and avoid artificial relationships from symptom similarity in our network analysis. A significance threshold of 0.25 and a *P* value<.01 determined statistical significance [24].

#### Network Estimation

We developed 3 networks for our study: an IADL network for all 5 IADL domains, a cognitive network for all 6 cognitive domains, and a bridge network linking both. Each network consisted of nodes (items within each domain) and edges (relationships between items). For the cognition network with continuous data, we applied the EBICglasso method, which used the Extended Bayesian Information Criterion (EBIC) with the least absolute shrinkage and selection operator (LASSO) for partial correlation analysis, reducing confounding by shrinking coefficients and zeroing smaller correlations. The IADL network, based on binary data, was analyzed using the IsingFit method, which used logistic regression to adjust node states and determine conditional probabilities. The bridge network was assessed using the “mgm” method, designed for mixed data types, using conditional independence tests tailored to heterogeneous data. Network visualization was performed using R packages *bootnet* and *qgraph*, where edge thickness represented association strength—blue for positive and red for negative associations [25].

#### Network Stability

The *bootnet* package was used to assess edge and centrality stability in each network [25]. Edge stability was evaluated through nonparametric bootstrap, with 95% CIs reflecting accuracy; narrower CIs indicate higher network reliability [25]. Centrality stability was measured with a case-dropping subset bootstrap, as reflected by the correlation stability coefficient (CS-C); values above 0.25, ideally over 0.5, denote optimal stability [25].

#### Central Node, Centrality, Bridge Node, and Bridge Centrality

A central node in a network has substantial influence due to its extensive connections with other nodes [26]. Bridge nodes connect different communities or clusters within a network, facilitating interactions that would otherwise not occur [27]. Centrality measures in network analysis typically include strength, betweenness, closeness, and expected influence; however, due to the instability of betweenness and closeness, and because strength ignores negative edges (summing only absolute values), we exclusively used expected influence for central nodes and bridge expected influence for bridge nodes [28]. The expected influence index, which accounts for both positive and negative edge values, was calculated using the *qgraph* package in R [26,27]. Similarly, the top bridge node was identified through the highest bridge expected influence (1-step) index, which sums the edge values connecting the node to those outside its community, determined by the *networktools* package in R [26,27]. Differences in node centrality were analyzed using Wilcoxon tests with 1000 bootstrapped indices from the bootnet package in R, applying Benjamini-Hochberg corrections for multiple comparisons [29].

#### Network Comparison Test

To analyze gender differences across 3 networks, we used the *network comparison test* package in R. This involved performing both a network invariance test, which identified significant variations in edges among subgroup networks, and a global strength invariance test, which evaluated the total weighted sum of all edges to measure the connection strength among network variables. Should the network invariance test yield significant results, we then conducted an edge invariance test to pinpoint specific edge pairs that varied between subgroups. In addition, we compared node centrality between men and women. To adjust for multiple comparisons at the level of individual edges and centralities, the Benjamini-Hochberg correction method was used.

### Ethical Considerations

This secondary analysis of the NHATS dataset relies on publicly available data. The original data collection, which obtained informed consent from all participants, was approved by the Johns Hopkins University IRB. As no restricted data were used, further IRB review was not required.

## Results

### Sample Characteristics and Descriptions of IADL and Cognitive Domains

Of the 2239 participants (1194 from wave 12 and 245 from wave 11), 1263 were female (56.41%), and 1720 (76.82%) were White. The predominant age group was 75‐79 years, representing 748 participants (33.41%). Detailed sociodemographic data, as well as descriptions and abbreviations of the IADL items and cognitive domains, are provided in Table 1.

**Table 1.** Demographics, descriptions, and abbreviations of instrumental activities of daily living (IADL) and cognitive domain items (N=2239).

Variables	Results	Range
Demographics, n (%)		
Sex		
Female	1263 (56.41)	—^a^
Male	976 (43.59)	—
Age group		
70‐74 years	214 (9.56)	—
75‐79 years	748 (33.41)	—
80‐84 years	620 (27.69)	—
85‐89 years	395 (17.64)	—
90+ years	262 (11.70)	—
Self-rated health		
Poor	69 (3.08)	—
Fair	431 (19.25)	—
Good	893 (39.88)	—
Very good	676 (30.19)	—
Excellent	169 (7.55)	—
Missing value	1 (0.04)	—
Race		
Non-Hispanic others	41 (1.83)	—
Non-Hispanic Black	375 (16.75)	—
Hispanic	81 (3.62)	—
Non-Hispanic White	1720 (76.82)	—
Missing	22 (0.98)	—
Living arrangement		
Alone	809 (36.13)	—
Living with someone	1430 (63.87)	—
Income status		
Poverty	859 (38.37)	—
Low income	413 (18.45)	—
Normal	967 (43.19)	—
Description of items (abbreviations in networks), n (%)		
Difficulty in managing medication (I1)		
No	1798 (80.30)	—
Yes	441 (19.70)	—
Difficulty in managing laundry (I2)		
No	1321 (59)	—
Yes	918 (41)	—
Difficulty in managing shopping (I3)		
No	1214 (54.22)	—
Yes	1025 (45.78)	—
Difficulty in managing meal (I4)		
No	1373 (61.32)	—
Yes	866 (38.68)	—
Difficulty in managing banking (I5)		
No	1505 (67.22)	—
Yes	734 (32.78)	—
Episodic memory (C1), mean (SD)	10.15 (3.21)	0-9
Executive function (C2), mean (SD)	0.84 (0.89)	0-5
Orientation (C3), mean (SD)	0.96 (1.33)	0-8
Psychomotor function (C4), mean (SD)	2.66 (0.13)	2.36-3.26
Visual attention (C5), mean (SD)	2.82 (0.10)	2.57-3.41
Working memory (C6)	0.34 (0.25)	0-1.57

a Not applicable.

### Items Remained After Redundancy Check

The Goldbricker analysis confirmed no redundancy in the IADL and cognitive domains, thus all items were retained.

### Stability of IADLs, Cognition, and the Bridge Networks

The bootstrapped 95% CI analysis revealed narrow CIs across all 3 networks (IADL, cognition, and bridge), indicating precise edge-weight estimates (Figures S1, S3, and S5 in Multimedia Appendix 1). In addition, CS-C values for the IADL, cognition, and bridge networks were 0.67, 0.75, and 0.44, respectively, all surpassing the recommended threshold of 0.25, confirming the networks’ interpretability and reliability (Figures S2, S4, and S6 in Multimedia Appendix 1).

### IADL Network

**Figure 1.**
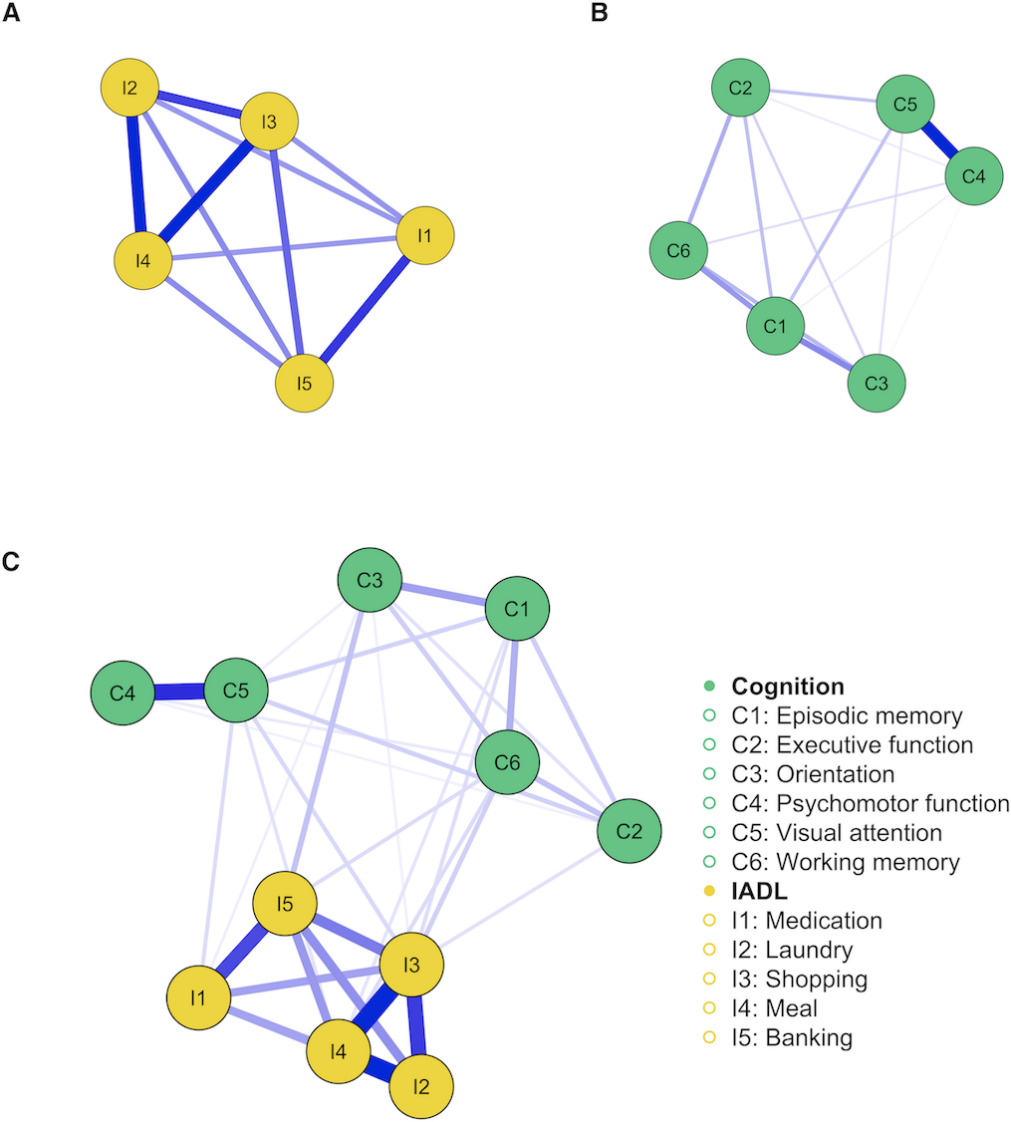
Network structure of instrumental activities of daily living (IADL) network, cognition network, and the bridge network.

**Figure 2.**
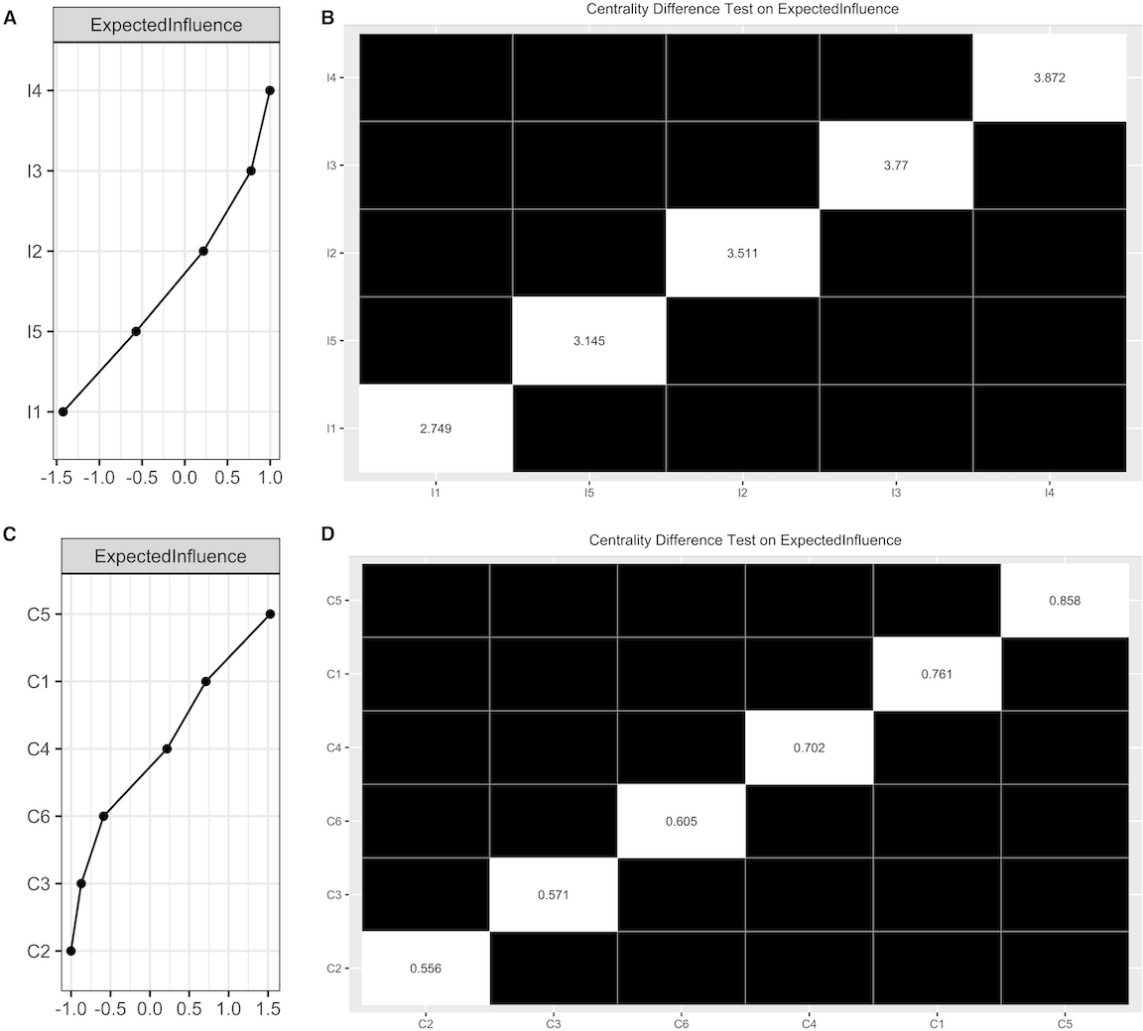
Expected influence centrality index and centrality bootstrapped difference tests for variables in the IADL network (Pane A and Panel B) and the cognition network (Panel C and Panel D). Gray boxes indicate variables that do not significantly differ from one-another. Black boxes represent variables that differ significantly from one another (α=.05). White boxes show the values of bridge expected influence. I1: difficulty in managing medication; I2: difficulty in managing laundry; I3: difficulty in managing shopping; I4: difficulty in managing meals; I5: difficulty in managing banking; C1: episodic memory; C2: executive function; C3: orientation; C4: psychomotor function; C5: visual attention; C6: working memory.

Figure 1A illustrates the IADL network, with all edges (10/10, 100%) nonzero, indicating strong connectivity among nodes. The most robust connections were between I3 and I4 (I3: shopping-I4:meal, edge weight 1.38), I2 and I4 (I2: laundry- I4:meal, 1.33), and I1 and I5 (I1: medication- I5: banking, 1.08). Logistic regression coefficients for other edges are detailed in Table S1 in Multimedia Appendix 1. Figure 2A shows that node I4 (meal) had the highest expected influence of 3.87. Figure 2B’s centrality bootstrapped difference test highlights I4’s (meal’s) significantly higher influence (all *P*<.05 after Benjamini-Hochberg corrections), underscoring its central role in the network.

### Cognition Network

Figure 1B shows the cognition network structure, with 14/15 edges (93.33%) nonzero, reflecting strong connectivity among nodes. The strongest connections were C4-C5 (C4: psychomotor function-C5: visual attention; edge weight 0.54), C1-C3 (C1: episodic memory-C3: orientation, 0.25), and C1-C6 (C1: episodic memory-C6: working memory, 0.22). The partial correlation matrix for other edges is in Table S2 in Multimedia Appendix 1. Figure 2C displays the expected influence index for all nodes, with C5 (visual attention) having the highest at 0.86. Figure 2D’s centrality bootstrapped difference test underscores C5’s (visual attention’s) significant influence within the cognitive domains (all *P*<.05 after Benjamini-Hochberg corrections).

### Bridge Network

**Figure 3.**
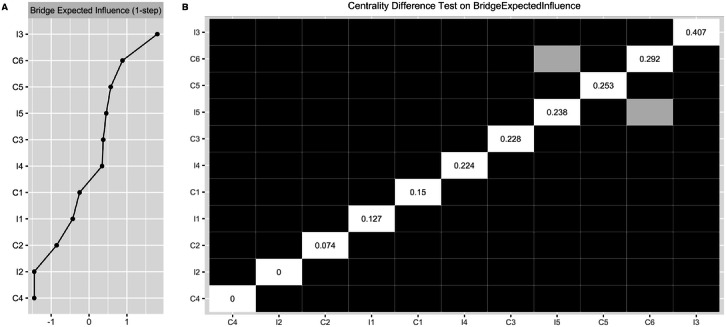
Bridge expected influence centrality index (Panel A) and centrality bootstrapped difference tests (Panel B) for variables in the bridge network. Gray boxes indicate variables that do not significantly differ from one-another. Black boxes represent variables that differ significantly from one another (α=.05). White boxes show the values of bridge expected influence. I1: difficulty in managing medication; I2: difficulty in managing laundry; I3: difficulty in managing shopping; I4: difficulty in managing meals; I5: difficulty in managing banking; C1: episodic memory; C2: executive function; C3: orientation; C4: psychomotor function; C5: visual attention; C6: working memory.

Figure 1C shows the bridge network between IADL and cognition, with 34/55 edges (61.82%) nonzero, indicating strong connectivity. Details on all edges are in Table 3 in Multimedia Appendix 1. Figure 3A highlights the bridge expected influence index, with I3 (shopping) recording the highest at 0.41, followed by I5 (banking, 0.24) and I4 (meal, 0.22). Significant bridge edges include I3-C6 (I3: shopping-C6: working memory, edge weight: 0.12), I5-C3 (I5: banking-C3: orientation, edge weight: 0.15), and I4-C6 (I4: meal-C6: working memory, edge weight: 0.09). Figure 3B’s centrality bootstrapped difference test confirms I3’s (shopping’s) prominent role in connecting IADL and cognitive domains (all *P*<.05 after Benjamini-Hochberg corrections).

### Sex Differences in Networks

Network invariance and global strength tests revealed no significant sex differences in the cognition network (*M*=0.07, *P*=.64; *S*=0.10, *P*=.21). Conversely, significant sex differences were evident in the IADL and bridge networks, confirmed by network invariance tests (IADL: *M*=0.95; *P*=.005 and bridge: *M*=0.49; *P*=.002) and global strength tests (IADL: *S*=1.94; *P*=.001 and bridge: *S*=1.79; *P*=.002). Following these findings, further edge and centrality invariance tests were conducted for the IADL and bridge networks between sexes. In the IADL network, edge invariance tests identified significant sex disparities between I2 (laundry) and I4 (meal), with females demonstrating stronger associations (female 1.69, male 0.74; *P*=.001) and greater centrality in I4 (meal: difference=1.99; *P*=.007). However, in the bridge network, no significant sex differences were detected through further edge and centrality invariance tests (all *P*>.05 after Benjamini–Hochberg corrections).

## Discussion

### Principal Findings

Using network analysis, the main findings of this study elucidates the detailed interactions within and between IADL and cognitive domains in older adults, identifying 3 key variables as predictive markers and potential intervention targets for enhancing global IADL and cognitive function, while also noting sex differences. First, meal preparation difficulty and visual attention are central nodes within their respective IADL and cognition networks, with higher levels predictive of better functionality; targeted modifications could significantly improve overall functionality. Secondly, shopping difficulty within the IADL network has the strongest association with global cognition. Given the cause-and-effect relationship between IADL and cognitive function, early interventions targeting shopping difficulties could effectively boost global cognition. Finally, a sex difference was observed, with meal preparation exerting a greater influence in the IADL network among females than males, suggesting its higher predictive relevance and intervention efficacy for females.

Within the IADL network, the biggest 3 connections include those between shopping and meal preparation, laundry and meal preparation, and medication management and banking. The strong link between shopping and meal preparation is due to their shared planning, organization, and physical demands necessary for food tasks [30-32]. Likewise, laundry and meal preparation share organizational and physical demands [13]. The link between medication management and banking stems from their reliance on the same executive functions and working memory [33,34]. Importantly, meal preparation is central in the IADL network, perhaps due to impacts on other domains like medication, shopping, and banking owning to necessary executive skills such as multitasking and planning [35,36]. Data from our cross-sectional analysis indicate that meal preparation is the most central node within the IADL network. This finding suggests that same unite improvement in meal preparation ability can yield the largest overall enhancement in IADL performance relative to other tasks. Therefore, targeted interventions—such as the integration of assistive kitchen technologies or cognitive orthoses—may be especially effective in promoting independent cooking and, by extension, broader functional independence [37,38].

In the cognition network, the strongest connections are between psychomotor function and visual attention, and between episodic memory, orientation, and working memory. The link between psychomotor and visual attention is due to their roles in coordinated, rapid response tasks [14]. Significant correlations also exist between episodic memory and gray matter volume in the bilateral hippocampus and parahippocampal gyrus, which are key for orientation [39]. Furthermore, the association between episodic memory and working memory is supported by evidence that working memory capacity and prefrontal cortex executive functions are essential for episodic memory formation and retrieval [40]. Importantly, visual attention, the most central node in the cognitive network, is crucial for selective focus necessary for advanced cognitive processes like reasoning and problem-solving [41], and it enhances complex task execution through interactions with working memory [42]. Studies show that enhancing visual attention can lead to sustained improvements in cognitive performance [43,44], supporting its pivotal role in overall cognition and highlighting the need for targeted interventions to enhance focus and processing of visual stimuli. Potential interventions include computer-based training programs, video games, mindfulness exercises, and virtual reality applications specifically designed for visual tracking tasks, all of which may contribute to enhanced cognitive function [45-48].

In the bridge network, which integrates both IADLs and cognition, connectivity is notably high, with 61.82% (34/55) of edges being nonzero. This finding indicates a robust interconnection among multiple cognitive domains and IADLs, suggesting that daily tasks rely on a synergy of cognitive processes rather than on any single discrete skill [49,50]. Such an observation aligns with mounting evidence that real-life cognition operates as an integrated set of processes-often termed functional cognition [51,52]. While traditional neuropsychological models emphasize isolated cognitive constructs, functional cognition highlights how domains such as attention, executive function, and memory converge to support everyday activities [53]. By adopting this framework, our findings on the specific links (“edges”) between each cognitive domain and each IADL can offer theoretical guidance for real-world functional cognition rehabilitation or training aimed at improving IADL performance. Importantly, the prominent bridge edge was identified between shopping and working memory. When considering the overall impact on global cognition, shopping ranked first as the bridge IADL, followed by banking and meal preparation. These latter activities are also highly demanding cognitively and should be targeted in interventions. However, if resources are limited, prioritizing shopping may yield the greatest benefits in enhancing overall cognitive function. Notably, shopping functions as a bridge IADL because it draws on a broad range of cognitive skills: episodic memory for recalling past purchases and layouts; executive function for planning and budgeting; spatial orientation for navigation; psychomotor skills for handling products and carts; visual attention for identifying items; and working memory for tracking purchases and costs [33,54-57]. To leverage its bridge position, shopping tasks could be integrated into routine cognitive assessments, and regular shopping activities could be encouraged to maintain cognitive function. Virtual reality simulations of shopping tasks, artificial intelligence-powered service robotics, and the use of audio recorders as assistive technology to enhance shopping independence among older adults [58,59], can leverage the bridge IADL role of shopping to effectively improve global cognitive function.

Sex differences within the IADL network show females with a stronger association between laundry and meal preparation and a higher centrality of meal preparation. These patterns likely result from societal norms assigning women more IADL responsibilities, especially laundry and meal preparation [17,60,61]. The elevated centrality of meal preparation in women’s IADL networks suggests a ripple effect where challenges in meal preparation deplete time management, mental energy, and physical resources, reducing efficiency in other tasks. This ripple effect also explains the stronger association between meal preparation and laundry among women compared with men. Previous studies indicate that IADLs do not measure equivalently for men and women [61]; our study finds that meal preparation has a lower predictive value for overall IADL function in males than in females. Interventions to improve IADL performance through meal preparation should be tailored with an awareness that these activities vary in importance and difficulty between sexes.

### Limitations

Several limitations are worth mentioning. First, the assessment of IADL relied on self-reported data, which, while expedient, may compromise reliability and necessitate cautious interpretation of the findings. Future research should use more objective measures to validate these results. Second, the cross-sectional design precludes the establishment of causality and does not capture the temporal dynamics between IADL capabilities and cognitive function, underscoring the need for longitudinal approaches. Third, we excluded older adults with dementia, as dementia-related deficits may mask subtle variations in both cognition and IADL performance, which is critical for identifying central or bridge nodes in network analysis. Consequently, our findings should be interpreted primarily for community-dwelling older adults with relatively preserved cognitive function. Future research should incorporate participants with more severe cognitive impairments to validate whether our findings remain consistent. Fourth, although we set eligibility at ≥65 years, all final participants were aged 70+ years, likely because those aged 65‐69 years in earlier waves either dropped out or turned 70 years old by Waves 11‐12. This may limit the generalizability of our findings to younger older adults. Fifth, our study relies on the NHATS dataset, which limits the assessment to a select set of IADL and cognitive domains. Sixth, one limitation of our study is that the network analysis did not adjust for external confounders (eg, age and cultural background). Network models focus solely on the relationships among the included nodes and do not account for factors outside the network. Future research might address this limitation by residualizing each node on confounders before constructing the network, or by using subgroup or multigroup analyses to examine how these factors influence network structure. While these measures capture key aspects of functioning, we acknowledge that not including additional, more nuanced domains may affect the generalizability of our findings. Finally, the generalizability of our findings is limited to the American population studied; it remains unclear if these results can be generalized to populations with differing cultural, economic, or health system backgrounds. Further studies should expand the demographic scope to determine if these findings hold across diverse populations.

### Conclusions

The central IADL, central cognitive domain, and bridge IADL connecting global cognition were meal preparation, visual attention, and shopping, respectively, underscoring the need for targeted interventions to maximize resource efficiency and effectiveness. Specifically, enhancing meal preparation in older adults may significantly boost holistic IADL capabilities through interventions such as cooking classes, nutritional education, and tailored tools, along with support services such as interdisciplinary collaboration, caregiver training, and smart appliances. Similarly, focusing on visual attention training through methods such as computer-based programs, neurofeedback, and mindfulness exercises may substantially improve global cognitive function. Given the link between IADL performance and cognitive function, interventions centered on shopping are expected to be highly effective. This can be achieved by integrating shopping tasks into cognitive assessments and promoting regular shopping activities. Technological aids such as GPS, virtual reality simulations, and caregiver education on the cognitive benefits of shopping can further support elderly care and quality of life. In addition, observed sex differences suggest that meal preparation interventions may vary in effectiveness, with potentially lower efficacy among males, highlighting the need for tailored strategies to maximize outcomes.

## Supplementary material

10.2196/67632 Multimedia Appendix 1 Tables S1-S3 and Figures S1-S6.

## Abbreviations

ASPE HHS: Office of the Assistant Secretary for Planning and Evaluation at the US Department of Health and Human Services
CS-C: correlation stability coefficient
EBIC: Extended Bayesian Information Criterion
FPL: Federal Poverty Level
IADL: instrumental activity of daily living
LASSO: least absolute shrinkage and selection operator
NHATS: National Health and Aging Trends Study
