# Supplementary material for: Network Analysis of Key Instrumental Activities of Daily Living and Cognitive Domains for Targeted Intervention in US Older Adults Without Dementia: Cross-Sectional Study
Source: JMIR Aging. 2025 Mar 19;8:e67632. doi: 10.2196/67632 (PMC11941277; doi:10.2196/67632)
Supplement: Multimedia Appendix 1 [file aging-v8-e67632-s001.docx]

## Multimedia Appendix 1: **Tables S1-S3 and Figures S1-S6**

|  | **Page number** |
| --- | --- |
| **Table S1**. Logistic regression coefficients matrix of variables in IADL network. | 2 |
| **Table S2.** Partial correlation matrix of variables in the cognition network. | 3 |
| **Table S3.** Conditional dependency coefficients matrix of variables in bridge network of cognition and IADL. | 4 |
| **Figure S1.** Bootstrapped confidence intervals of edge weights for the IADL network. | 5 |
| **Figure S2.** The stability of expected influence centrality index in IADL network using case-dropping bootstrap. | 6 |
| **Figure S3.** Bootstrapped confidence intervals of edge weights for the cognition network. | 7 |
| **Figure S4.** The stability of expected influence centrality index in cognition network using case-dropping bootstrap. | 8 |
| **Figure S5.** Bootstrapped confidence intervals of edge weights for the bridge network. | 9 |
| **Figure S6**. The stability of expected influence centrality index in the bridge network using case-dropping bootstrap. | 10 |

**Table S1.** Logistic regression coefficients matrix of variables in IADL network.

|  | I1 | I2 | I3 | I4 | I5 |
| --- | --- | --- | --- | --- | --- |
| I1 | 0.00000 | 0.54985 | 0.57247 | 0.54953 | 1.07714 |
| I2 | 0.54985 | 0.00000 | 1.00327 | 1.33237 | 0.62552 |
| I3 | 0.57247 | 1.00327 | 0.00000 | 1.37080 | 0.82325 |
| I4 | 0.54953 | 1.33237 | 1.37080 | 0.00000 | 0.61921 |
| I5 | 1.07714 | 0.62552 | 0.82325 | 0.61921 | 0.00000 |

IADL: instrumental activities of daily living; I1: difficulty in managing medication; I2: difficulty in managing laundry; I3: difficulty in managing shopping; I4: difficulty in managing meals; I5: difficulty in managing banking.

**Table S2.** Partial correlation matrix of variables in the cognition network.

|  | C1 | C2 | C3 | C4 | C5 | C6 |
| --- | --- | --- | --- | --- | --- | --- |
| C1 | 0.00000 | 0.13136 | 0.25210 | 0.03287 | 0.12718 | 0.21723 |
| C2 | 0.13136 | 0.00000 | 0.08994 | 0.04340 | 0.12321 | 0.16785 |
| C3 | 0.25210 | 0.08994 | 0.00000 | 0.01321 | 0.06367 | 0.15244 |
| C4 | 0.03287 | 0.04340 | 0.01321 | 0.00000 | 0.54432 | 0.06779 |
| C5 | 0.12718 | 0.12321 | 0.06367 | 0.54432 | 0.00000 | 0.00000 |
| C6 | 0.21723 | 0.16785 | 0.15244 | 0.06779 | 0.00000 | 0.00000 |

C1: episodic memory; C2: executive function; C3: orientation; C4: psychomotor function; C5: visual attention; C6: working memory.

**Table S3.** Conditional dependency coefficients matrix of variables in bridge network of cognition and IADL.

|  | C1 | C2 | C3 | C4 | C5 | C6 | I1 | I2 | I3 | I4 | I5 |
| --- | --- | --- | --- | --- | --- | --- | --- | --- | --- | --- | --- |
| C1 | 0.00000 | 0.12429 | 0.23619 | 0.00000 | 0.11146 | 0.19650 | 0.00000 | 0.00000 | 0.08443 | 0.06533 | 0.00000 |
| C2 | 0.12429 | 0.00000 | 0.07263 | 0.03177 | 0.11374 | 0.15302 | 0.00000 | 0.00000 | 0.07402 | 0.00000 | 0.00000 |
| C3 | 0.23619 | 0.07263 | 0.00000 | 0.00000 | 0.04299 | 0.12189 | 0.04117 | 0.00000 | 0.03493 | 0.00000 | 0.15186 |
| C4 | 0.00000 | 0.03177 | 0.00000 | 0.00000 | 0.53138 | 0.05342 | 0.00000 | 0.00000 | 0.00000 | 0.00000 | 0.00000 |
| C5 | 0.11146 | 0.11374 | 0.04299 | 0.53138 | 0.00000 | 0.00000 | 0.08618 | 0.00000 | 0.09623 | 0.07043 | 0.00000 |
| C6 | 0.19650 | 0.15302 | 0.12189 | 0.05342 | 0.00000 | 0.00000 | 0.00000 | 0.00000 | 0.11712 | 0.08840 | 0.08625 |
| I1 | 0.00000 | 0.00000 | 0.04117 | 0.00000 | 0.08618 | 0.00000 | 0.00000 | 0.21803 | 0.23444 | 0.23164 | 0.45578 |
| I2 | 0.00000 | 0.00000 | 0.00000 | 0.00000 | 0.00000 | 0.00000 | 0.21803 | 0.00000 | 0.49442 | 0.64320 | 0.27696 |
| I3 | 0.08443 | 0.07402 | 0.03493 | 0.00000 | 0.09623 | 0.11712 | 0.23444 | 0.49442 | 0.00000 | 0.62334 | 0.33039 |
| I4 | 0.06533 | 0.00000 | 0.00000 | 0.00000 | 0.07043 | 0.08840 | 0.23164 | 0.64320 | 0.62334 | 0.00000 | 0.25725 |
| I5 | 0.00000 | 0.00000 | 0.15186 | 0.00000 | 0.00000 | 0.08625 | 0.45578 | 0.27696 | 0.33039 | 0.25725 | 0.00000 |

IADL: instrumental activities of daily living; I1: difficulty in managing medication; I2: difficulty in managing laundry; I3: difficulty in managing shopping; I4: difficulty in managing meals; I5: difficulty in managing banking; C1: episodic memory; C2: executive function; C3: orientation; C4: psychomotor function; C5: visual attention; C6: working memory.

**Figure S1.** Bootstrapped confidence intervals of edge weights for the IADL network.


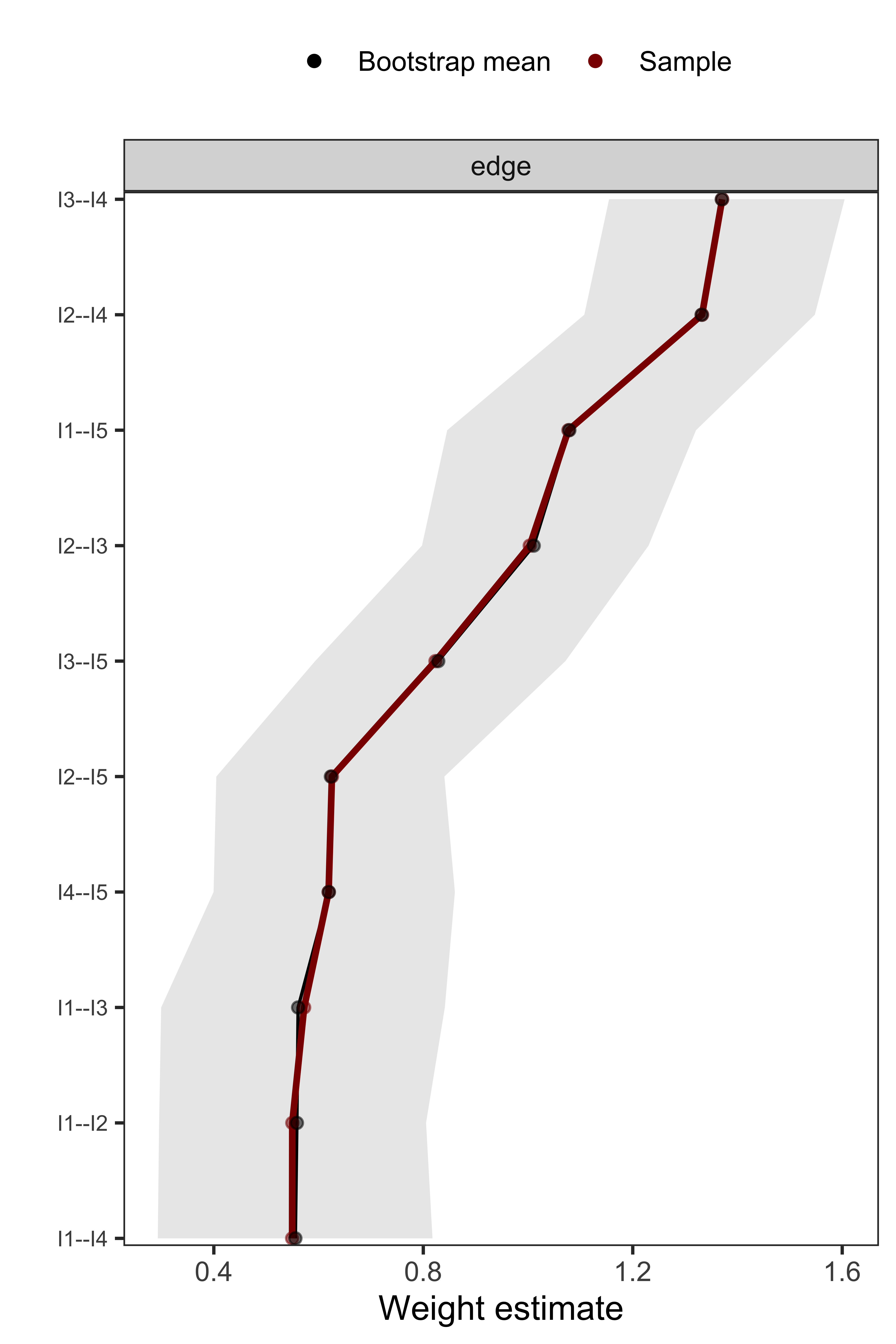


The red dots are sample means per edge, while the black dots are bootstrapped means, ordered from the highest to the lowest value. The gray area represents the 95% confidence intervals of edge weights, estimated with the non-parametric bootstrap procedure (Bootnet package). Wide intervals indicate lower stability and narrow intervals indicate higher stability. IADL: instrumental activities of daily living; I1: difficulty in managing medication; I2: difficulty in managing laundry; I3: difficulty in managing shopping; I4: difficulty in managing meals; I5: difficulty in managing banking.

**Figure S2.** The stability of expected influence centrality index in the IADL network using case-dropping bootstrap.


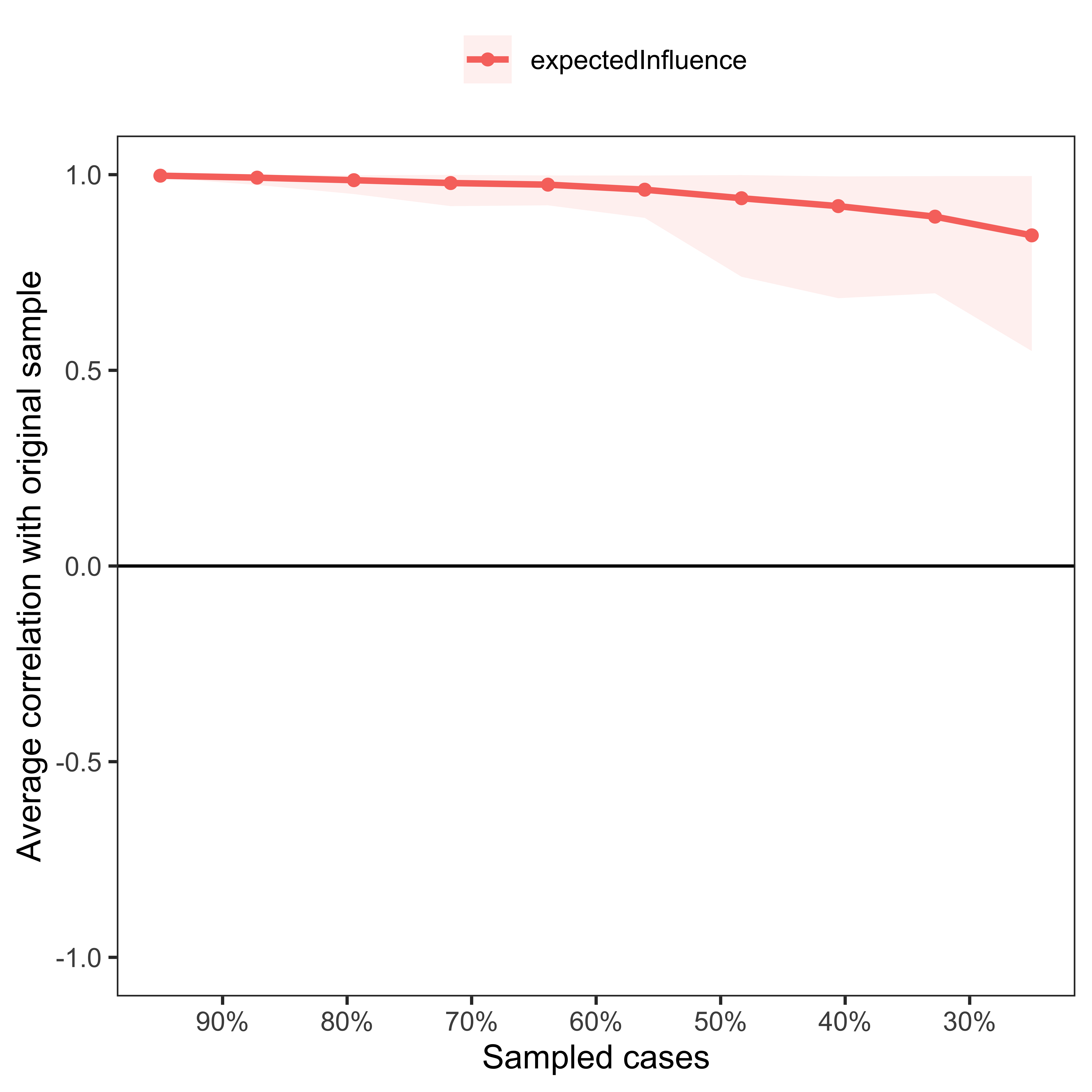


The x-axis indicates the percentage of cases of the original sample included at each step. The y-axis indicates the average of correlations between the expected influence centrality index from the original network and the expected influence centrality index from the networks that were re-estimated after excluding increasing percentages of cases. IADL: instrumental activities of daily living

**Figure S3.** Bootstrapped confidence intervals of edge weights for the cognition network.


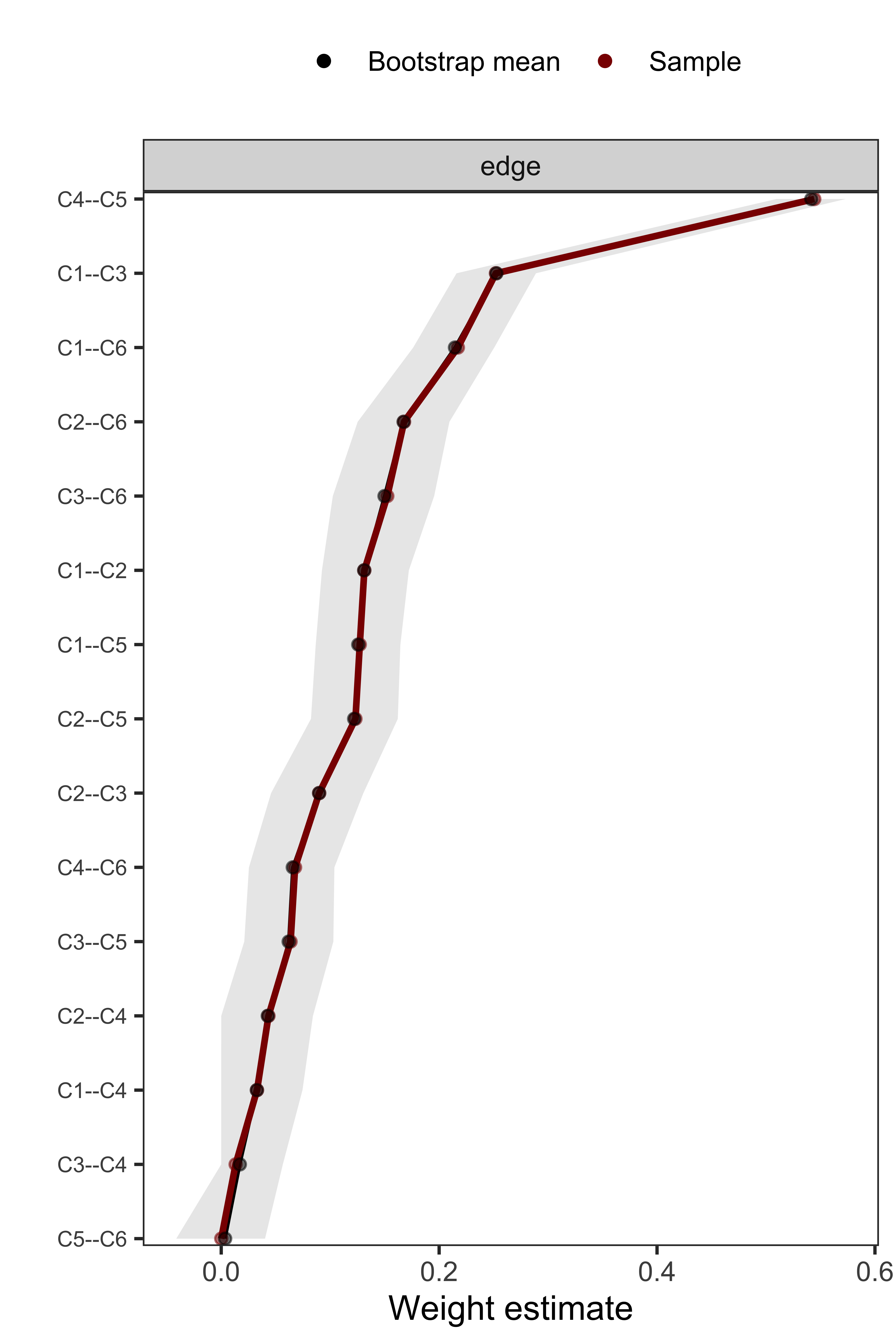


The red dots are sample means per edge, while the black dots are bootstrapped means, ordered from the highest to the lowest value. The gray area represents the 95% confidence intervals of edge weights, estimated with the non-parametric bootstrap procedure (Bootnet package). Wide intervals indicate lower stability and narrow intervals indicate higher stability. C1: episodic memory; C2: executive function; C3: orientation; C4: psychomotor function; C5: visual attention; C6: working memory

**Figure S4.** The stability of expected influence centrality index in the cognition network using case-dropping bootstrap.


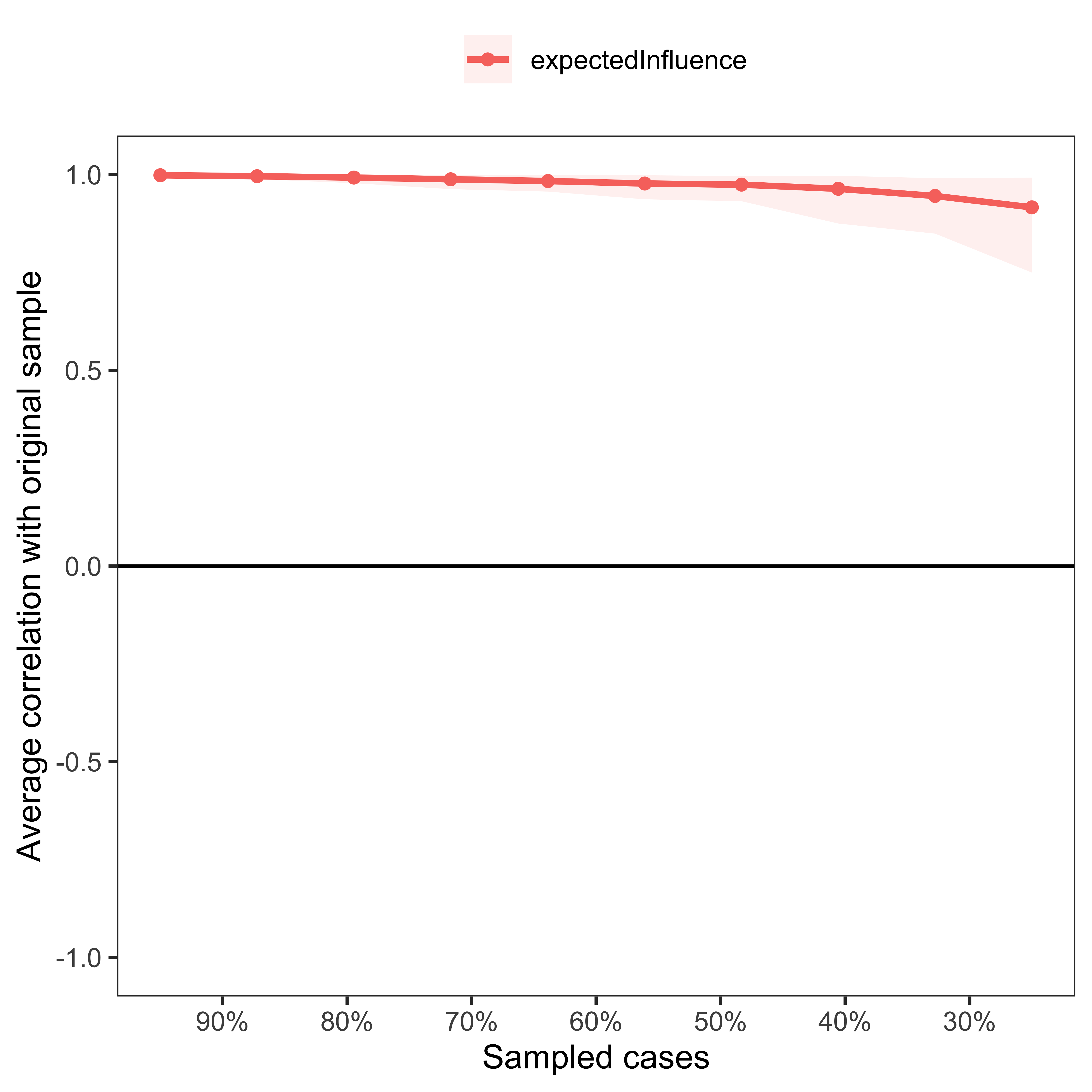


The x-axis indicates the percentage of cases of the original sample included at each step. The y-axis indicates the average of correlations between the expected influence centrality index from the original network and the expected influence centrality index from the networks that were re-estimated after excluding increasing percentages of cases.

**Figure S5.** Bootstrapped confidence intervals of edge weights for the bridge network.





The red dots are sample means per edge, while the black dots are bootstrapped means, ordered from the highest to the lowest value. The gray area represents the 95% confidence intervals of edge weights, estimated with the non-parametric bootstrap procedure (Bootnet package). Wide intervals indicate lower stability and narrow intervals indicate higher stability. I1: difficulty in managing medication; I2: difficulty in managing laundry; I3: difficulty in managing shopping; I4: difficulty in managing meals; I5: difficulty in managing banking; C1: episodic memory; C2: executive function; C3: orientation; C4: psychomotor function; C5: visual attention; C6: working memory

**Figure S6**. The stability of expected influence centrality index in the bridge network using case-dropping bootstrap.


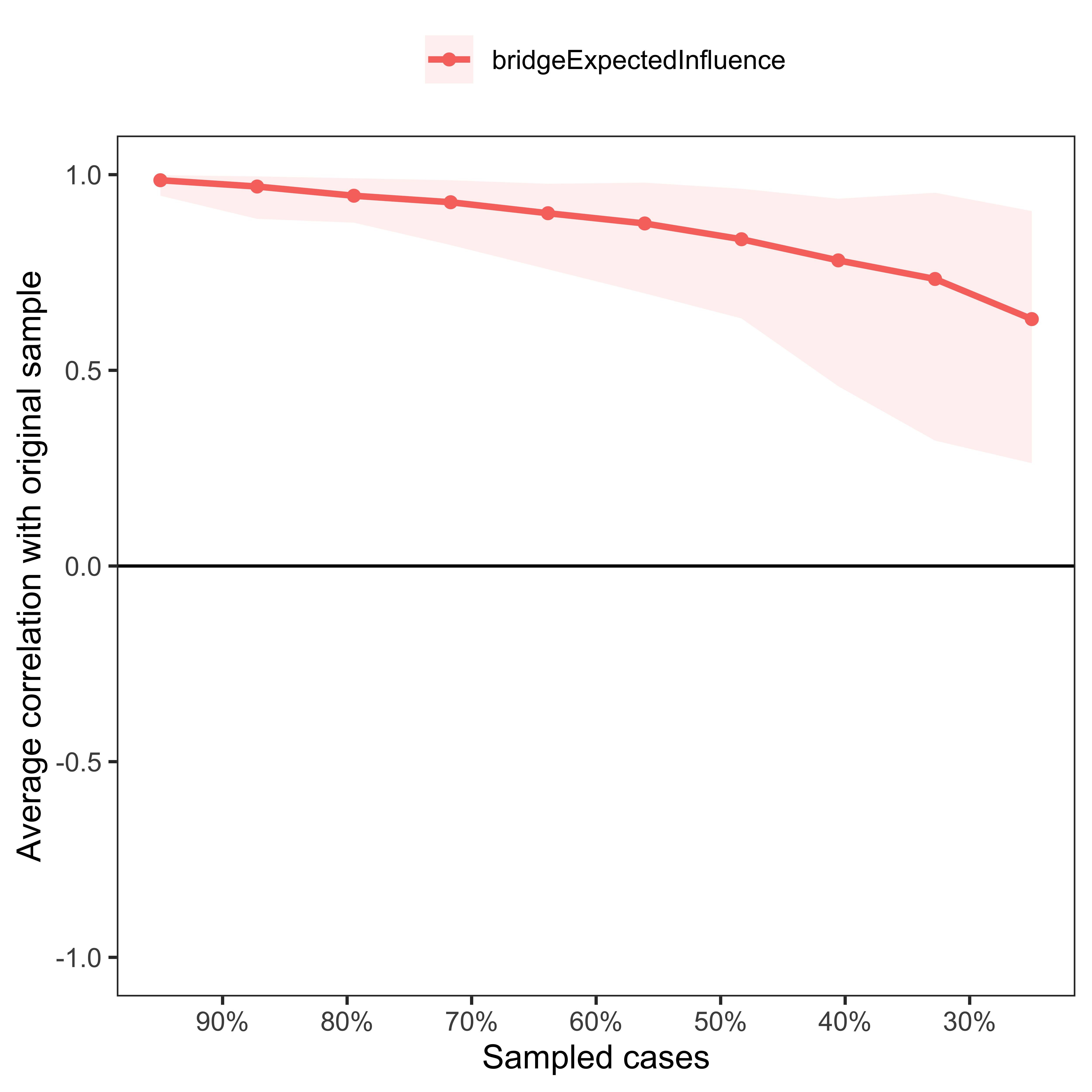


The x-axis indicates the percentage of cases of the original sample included at each step. The y-axis indicates the average of correlations between the bridge expected influence centrality index from the original network and the bridge expected influence centrality index from the networks that were re-estimated after excluding increasing percentages of cases.
